# A bibliometric analysis of autophagy in lung diseases from 2012 to 2021

**DOI:** 10.3389/fimmu.2022.1092575

**Published:** 2022-12-16

**Authors:** Feihong Lin, Yong Chen, Wei Mo, Huanping Zhou, Zhuoran Xiao, Song Hu, Xuan Shi, Meiyun Liu, Juan Wei, Wanli Zhu, Sheng Wang, Xin Lv

**Affiliations:** Department of Anesthesiology, Shanghai Pulmonary Hospital, School of Medicine, Tongji University, Shanghai, China

**Keywords:** autophagy, lung, bibliometrics, bibliometrix, VOSviewer, citespace

## Abstract

**Background:**

Autophagy refers to the process in which cells wrap their damaged organelles or unwanted proteins into a double-membrane structure and direct them to lysosomes for degradation. Autophagy can regulate many lung diseases such as pulmonary hypertension, acute lung injury, and lung cancer. However, few bibliometric studies on autophagy are available. The aim of the present study was to clarify the role of autophagy in lung diseases by bibliometric analysis.

**Methods:**

Publications were retrieved from the 2012–2021 Science Citation Index Expanded of Web of Science Core Collection on 20 September 2022. Bibliometrix package in R software was used for data retrieval. VOSviewer and CiteSpace were used to visualize the research focus and trend regarding the effect of autophagy on lung disease.

**Results:**

A total of 4,522 original articles and reviews on autophagy in lung diseases published between 2012 and 2021 were identified. China had the largest number of published papers and citations, whereas the United States (US) ranked first in the H-index and G-index. Moreover, cooperation network analysis showed close cooperation between the US, China, and some European countries, and the top 10 affiliates were all from these countries and regions. Bibliometric analysis showed that “autophagy” and “apoptosis” were the keywords with the highest frequency. During the past decade, most studies were concerned with basic research on pathways related to the regulatory role of autophagy in the inhibition and attenuation of lung diseases.

**Conclusion:**

The study of autophagy in lung diseases is still in the development stage. The information published in these articles has helped researchers understand further the hot spots and development trends in the field more and learn about the collaboration network information regarding authors, countries, and institutions, as well as the paper citation correlation. More studies have been performed to gain deeper insights into the pathogenesis of autophagy by focusing on the links and effects between various diseases. More recently, research in this field has paid increasing attention to the function of autophagy in COVID-19–related lung diseases.

## Introduction

Autophagy (“self-eating” in Greek) refers to the process in which cells wrap their damaged organelles or unwanted proteins into a double-membrane structure and direct them to lysosomes for degradation (1, 2). Autophagy exhibits a strong conservative property in all eukaryotes and is essential for maintaining cellular homeostasis. Hence, nearly all cell types present a basal autophagy level. Normally, autophagy takes charge of regulating the physiological functions, degrading cellular components, and forming building blocks and cell response to stress (RTS) (2). Autophagy can be easily induced by environmental insults, and the autophagic cycle rate (flux) can commonly change in RTS (3). Upon most occasions, induction of autophagy in RTS is a cytoprotective mechanism. Despite this, undue self-degradation results in cell death (4–6). The lung is a complex organ mainly functioning in gas exchange and is composed by many cell types including endothelial, epithelial, inflammatory, and mesenchymal cell types (7). Autophagy is an inducible RTS in lung cells. Agents triggering autophagy present a special association with lung cell biology, including hypoxia, particle and cigarette smoke exposure, pro-inflammatory states, and conditions that promote endoplasmic reticulum (ER) stress or oxidative stress. An increasing number of lung-related diseases are emerging, such as chronic obstructive pulmonary disease (COPD), idiopathic pulmonary fibrosis, pulmonary hypertension, acute lung injury, and lung cancer, which form a crux in the field of malignancy (8), causing high mortality (9, 10). Although some progress has been made in research of the effect of autophagy on lung diseases, many issues need to be further explored.

Bibliometric analysis pays attention to the literature systems and literature characteristics and broadly serves for understanding the knowledge structure as well as exploring the developmental trends by virtue of the qualitative and quantitative scientific literature analysis (11). Bibliometric analysis quantitatively measures the study domain outline distribution as well as the relationship and clustering. It describes and predicts the future development of a certain research field on the one hand and compares the contributions made by different authors, institutions, countries, and journals on the other hand. As bibliometric analysis can help develop guidelines, understand research hot spots, and evaluate the research trends (12), it has been applied to the research of digestive system diseases (13), cancer (14), rheumatic system diseases (15), and nervous system diseases (16). However, there are basically no bibliometric studies investigating the autophagy in lung diseases. Hence, the aim of this study is to make a systematical analysis of the research on the autophagy in lung diseases and to evaluate the related research state and trend.

## Materials and methods

### Data sources and search strategies

**Figure 1:**
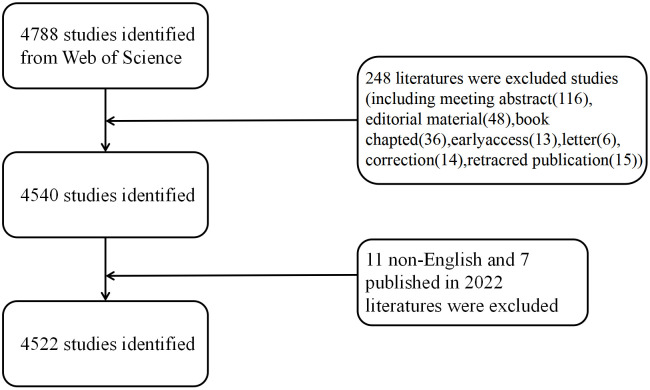
Detailed process for literature screening.

We performed a systematic search for the Science Citation Index Expanded (SCI-Expanded) of Web of Science Core Collection (WoSCC) in 2012–2021 during 1 January 2012 and 31 December 2021, and we downloaded the data on 20 September 2022 for avoiding deviations. The search terms included: TS = (autophagy) AND TS= (“pulmonary” OR “lung”). Two reviewers (FH Lin and JX Yuan) took charge of independently identifying this data search and discussing underlying differences, with the final agreement of 0.90 (17). After excluding the online publication time of 2022 and articles written in non-English and by limiting the publication type to reviews and original articles, we finally obtained 4,522 original articles and reviews that met the criteria for inclusion in the analysis (**Figure 1**).

### Data collection and cleaning

First, the original data were extracted from the SCI-Expanded database of WoSCC. The recorded information included the number of papers and citations, H-index, publication year, countries/regions, affiliations, authors, journals, references, and keywords. Afterward, duplicate authors and misspelled elements were moved artificially. Although inaccurate analysis may not be avoided completely due to multiple versions of cited references, the same abbreviated names of different authors, and different forms of cited journals, we pointed out the first extracted original data from the SCI-Expanded database. The recorded information was paper and citation number, H-index, G-index publication year, countries/regions, affiliations, authors, journal, references, and keywords. Then, we removed misspelled elements and duplicate authors. Cited references have different versions, different authors may have the same abbreviated name, and cited journals have different forms, which may lead to inaccurate analysis. Despite this, most raw data were accurate. We removed the misspelled elements and duplicate authors and adopted a thesaurus file for merging duplicates into one word, deleting useless words and correcting the misspelled elements. Later, we imported the cleansed data to VOSviewer (version 1.6.18.0), CiteSpace (version 6.1. R3), and the “bibliometrix package 4.0.1” of R software (version 4.2.1) for the subsequent bibliometric analysis.

### Bibliometric analysis

The number of papers and citations is the bibliometric indicator commonly used to represent the bibliographic material. In general, the research level can be evaluated from the perspective of productivity and impact, which, in the paper, were respectively measured *via* the number of publications (NP) and the number of citations without self-citations (NC). Sometimes, H-index is used to evaluate the individual academic achievements or the publication output (18). G-index refers to the highest number of papers that receive H-index or more citations (19). In addition, the article value can also be indicated by the impact factor (IF) from the latest version of Journal Citation Reports (20, 21). We visualized the results for the number of papers per year, country, organization, author, citation, and other relevant aspects. Prior to data analysis, the R4.2.1-based Bibliometrix package was used to store, count, and clean up data. As important indicators of the research, H-index, G-index, and IF were all included in the analysis. Subsequently, the following software was used for bibliometric analysis.

R (version 4.2.1) stands for the language and environment specific to the statistical computing and graphics. It presents a strong extensibility, capable of automating the analyses and creating new functions. The cleansed data received bibliometric analysis under the assistance of bibliometrix package in R (22). To further explain the annual document quantity alternation, we employed the fitting polynomial model for predicting the annual NP. Variable f (x) refers to the annual study number, and x stands for the publication year. Moreover, VOSviewer software assisted in constructing bibliometric maps for obtaining more extensive information regarding the results considering co-citation (CC) and co-occurrence (CO) (23, 24). Co-citation refers to the situation that two items are simultaneously cited by the third one. Keyword co-occurrence takes charge of measuring the keywords appearing in the same documents most frequently (25). Analysis on the keywords serves for demonstrating the autophagy-related research in lung disease.

VOSviewer, CiteSpace, and R (version 4.2.1) were used for the statistical computing and graphics. VOSviewer uses data from WoSCC for establishing the bibliometric maps (23), capable of assisting in comprehensively viewing the bibliometric maps in detail considering the collaborative data. CiteSpace focuses on analyzing the underlying knowledge in the scientific literature and visualizing the collected data (26).

## Results

### An overview of publications on autophagy in lung disease

The number of retrieved publications was 4,522, including 3,772 original research articles and 750 reviews, with total NC of 114,589, a mean NC/article ratio of 27.91, and a mean H-index of 130.

### The annual trend of paper publication quantity

**Figure 2:**
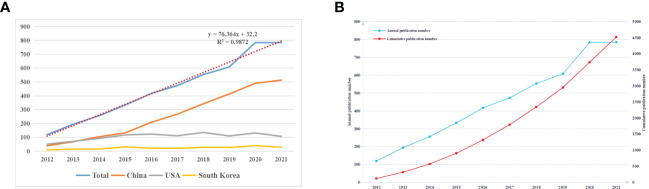
**(A)** The total NP and top three countries by number of published papers during 2012 and 2021. **(B)** The annual NP and accumulation during 2012 and 2021.

**Figure 2A** displays the annual NP associated with autophagy in lung diseases. The annual paper number indicates a fast increase from 119 in 2012 to 785 in 2021, and the NP reached the peak in 2021. From 2012, annual NP did not change significantly in the United States (US) and Korea but indicated a fast elevation in China, and there has been a surge since 2015. The annual NP exhibited an obvious relation to the publication year, with the correlation coefficient R^2^ of 0.9872 (**Figure 2A**). The rapidly increasing NP demonstrates the increasing number of scholars who placed emphasis on this field. There were more than 4,500 publications in 2021, demonstrating the increasing number of scholars who conducted relevant research and the flourish of lung disease-related autophagy research theories (**Figure 2B**).

### Contributions of countries/regions to global publications

**Figure 3:**
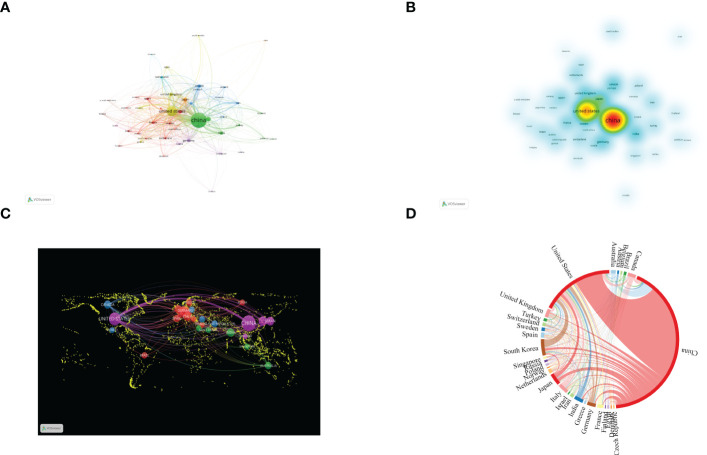
Contributions of various countries to the research of autophagy in lung diseases. **(A)** Country collaboration map of autophagy research in lung disease. Circles denote countries, and lines denote their collaborations. **(B)** Countries’ density map. **(C)** World map of countries’ cooperation density. **(D)** A circle diagram evaluating the international collaboration between clusters.

The 10 high-output countries/regions with the highest output were ranked according to NP of all authors. China published the most papers (2,564, 56.7%), followed by the US (1,048, 23.18%) and South Korea (234, 5.17%) (**Figures 3A, B**; **Table 1**). NC was 23,791 for China, accounting for 38.2% of the total number of citations, followed by the US (47,846) and South Korea (5,449). However, the US enjoyed the highest H-index (181) and G-index (264), about three times that of South Korea (58 of 81).

**Table 1:** Top 10 countries with the most published research on autophagy in lung disease.

Rank	Country/Region	NP	% of (4,522)	NC	H-index	G-index
1	China	2,564	56.70	54,251	118	163
2	United States	1,048	23.18	47,846	181	264
3	South Korea	234	5.17	5,449	58	81
4	Japan	169	3.74	5,943	69	102
5	Italy	148	3 27	6,054	75	105
6	India	132	2.92	3,488	43	72
7	Germany	126	2.79	4,740	62	102
8	United Kingdom	122	2.70	5,042	59	89
9	Canada	121	2 68	5,305	64	100
10	France	92	2.03	4,141	75	134

**Figures 3C, D** display the publication distribution in various countries and regions. Related cooperation was mainly concentrated upon China and the US, and cooperation with other countries was relatively weak. As shown in **Figure 3C**, cooperation between countries and regions significantly promoted the development of scientific research. The lines in the figure indicate cooperation: The wider the lines, the stronger the cooperation. Nevertheless, lines cannot be found in most countries, meaning that these countries had no stable communication and cooperation.

### Analysis of authors

**Figure 4:**
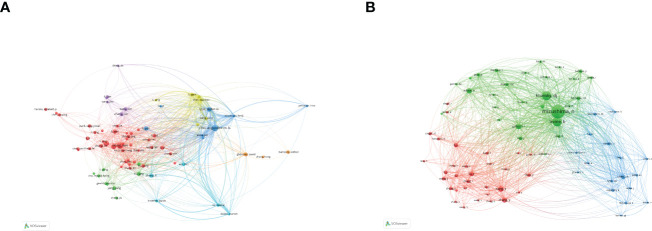
Authors related to the research on autophagy in lung disease. **(A)** Author co-occurrence. **(B)** VOSviewer visualization of co-cited authors. Circle size means the number of authors’ published articles; the connecting lines stand for authors’ mutual communication and interaction.

According to the author visualization chart by VOSviewer, 78 authors performed autophagy-related studies. In terms of the NP, Choi Augustine M. K. (28 publications) ranked first, followed by Ryter Stefan W. (28 publications), Wang Jing (25articles), and Wang Wei (24 articles) (**Figure 4A**; **Table 2**). Obviously, there was a relatively low author centrality (≤0.04), demonstrating the necessity to enhance the autophagy impact in lung disease. Co-cited authors refer to those (at least two) who are cited at the same time (**Figure 4B**). Only six had a frequency of citation of over 400 times amid the 103,587 co-cited authors. The lines between nodes stand for authors’ collaboration, and the collaboration graph means that authors’ collaboration indeed existed in the field.

**Table 2:** Top 10 authors and co-cited authors related to autophagy in lung disease.

Rank	Author	Count (%)	Centrality	Co-cited author	Citation	Centrality
1	Choi Augustine M. K.	28 (0.62%)	0.03	Mizushima N.	1,170	0.02
2	Wang Jing	25 (0.55%)	0.02	Levine B.	696	0.01
3	Wang Wei	24 (0.53%)	0.00	Klionsky D. J.	696	0.01
4	Ryter Stefan W.	21 (0.46%)	0.00	White E.	352	0.01
5	Zhang Wei	19 (0.42%)	0.02	Galluzzi L.	348	0.03
6	Li Yan	18 (0.40%)	0.02	Wang Y.	341	0.02
7	Liu Ying	14 (0.31%)	0.04	Zhang Y.	322	0.01
8	Ghavami Saeid	14 (0.31%)	0.00	Chen Z. H.	311	0.03
9	Li Yi	14 (0.31%)	0.02	Liu Y.	279	0.01
10	Zhang Li	14 (0.31%)	0.02	Kroemer G.	274	0.02

### Analysis of affiliations

**Figure 5:**
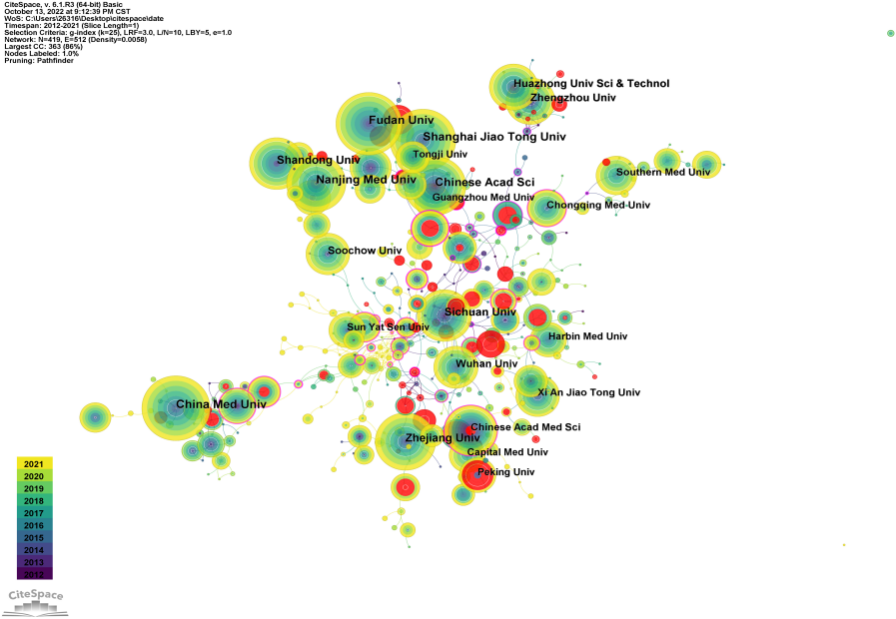
Visualization of institutions that performed research on autophagy in lung disease.

**Figure 5** is an institutional co-occurrence map built with CiteSpace, listing the top 10 institutions considering the output and centrality in autophagy-related lung disease studies. Nodes stand for institutions: The larger the node, the more output the institution made. Links between nodes mean institutional collaboration, and the color and thickness denote the collaboration duration and intensity, respectively. China Medical University (n = 190) was in the leading position considering the output, followed by Shandong University (n =180) and Zhejiang University (n = 174). The top three institutions considering the centrality were New York University (0.37), Pittsburgh University (0.30), and Michigan University (0.26) (**Table 3**). As the data show, institutions were closely collaborated with each other.

**Table 3:** Top 10 institutions conducting autophagy in lung disease by volume and centrality.

Rank	Affiliations	Count	Rank	Affiliations	Centrality
1	China Med Univ	190	1	New York Univ	0.37
2	Shandong Univ	180	2	Pittsburgh Univ	0.30
3	Zhejiang Univ	174	3	Michigan Univ	0.26
4	Fudan Univ	164	4	Lovelace Resp Res Inst	0.21
5	Nanjing Med Univ	157	5	UIsan Univ	0.21
6	Shang Hai Jiao Tong Univ	157	6	Brigham &Women’s Hosp	0.20
7	Sichuan Univ	143	7	Chinese Acad Med Sci	0. 19
8	Chongqing Med Univ	116	8	Seoul Natl Univ	0.19
9	Seoul Natl Univ	111	9	Baylor Coll Med	0.18
10	Huazhong Univ Sci and Technol	109	10	Roma Tor Vergata Univ	0.18

### Analysis of journals

**Figure 6:**
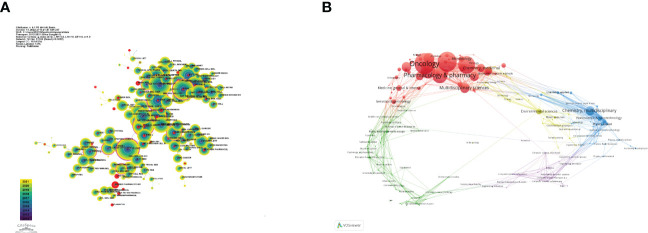
Visualization of co-cited journals on lung disease-related autophagy. **(A)** Circles denote co-cited journals; connecting lines stand for journal interaction. **(B)** Superposition analysis of research fields.

**Table 4** gives the top 10 journals considering the research number of lung disease–related autophagy and the impact indicators, namely, H-index, G-index, and IF eigenfactor score. These journals had a larger possibility in accepting articles regarding autophagy in lung disease, given their largest NP of relevant topics. Scholars in the autophagy area are suggested to place emphasis on these journals. ONCOTARGET (115 publications, IF = 4.147) had the highest NP, followed by INT J MOL SCI (98 publications, IF = 5.924), PLOS ONE (97 publications, IF = 3.248), CELL DEATH DIS (81 publications, IF = 8.468), and AUTOPHAGY (80 publications, IF = 16.016). Notably, the AUTOPHAGY had higher citations, H-index, and G-index, demonstrating that the quality of many studies was not high. In **Figure 6A**, the analysis of journals revealed that PLOS ONE (2,677 total citations) presented the highest citation frequency, followed by AUTOPHAGY (2,626 total citations) and J BIOL CHEM (2,440 citations). J BIOL CHEM (0.58) enjoyed the highest centrality, followed by PLOS ONE (0.40) and P NATL ACAD SCI USA (0.39) (**Table 5**), all of which had a high centrality, demonstrating their stronger impact in this field. In the domain dimension, superposition graph analysis was carried out for the fields involved in the literature (**Figure 6B**). Among them, the top fields of paper production were oncology, pharmacology and pharmacy, chemistry medicinal, multidisciplinary sciences, and chemistry multidisciplinary. Therefore, in the study of autophagy in lung diseases, oncology and pharmacochemistry are currently the focused areas.

**Table 4:** Top 10 journals for co-citation of autophagy research in lung disease.

Rank	Journal	NP	NC	H-index	G-index	IF (2020)
1	ONCOTARGET	115	3,605	35	51	4.147
2	INT J MOL SCI	98	3,351	24	56	5.924
3	PLOS ONE	97	3,491	36	54	3.240
4	CELL DEATH DIS	81	2,487	30	46	8.468
5	AUTOPHAGY	80	4,858	43	69	16.016
6	SCI REP-UK	80	2,013	26	40	4.380
7	BIOCHEM BIOPH RES CO	64	1,025	19	29	3.575
8	BIOMED PHARMACOTHER	55	1,204	20	32	6.529
9	FRONT PHARMACOL	54	903	18	28	5.811
10	ONCOL LETT	53	803	16	26	2.967

**Table 5:** Top 10 journals for centrality of autophagy research in lung disease.

Rank	Journal	Centrality	JCR	IF (2020)
1	J Biol Chem	0.58	Q2	5.1571
2	Plos One	0.40	Q2	3.204
3	P Natl Acad Sci Usa	0.39	Q1	11.205
4	Cancer Res	0.36	Q1	12.702
5	Am J Resp Crit Care	0.23	Q1	21.404
6	Cell	0.21	Q1	41.584
7	Clin Cancer Res	0.20	Q1	12.531
8	J Immunol	0.16	Q1	5.422
9	Int J Mol Sci	0.14	Q1	5.924
10	Free Radical Bio Med	0.11	Q1	7.376

JCR, Journal Citation Reports.

**Figure 7:**
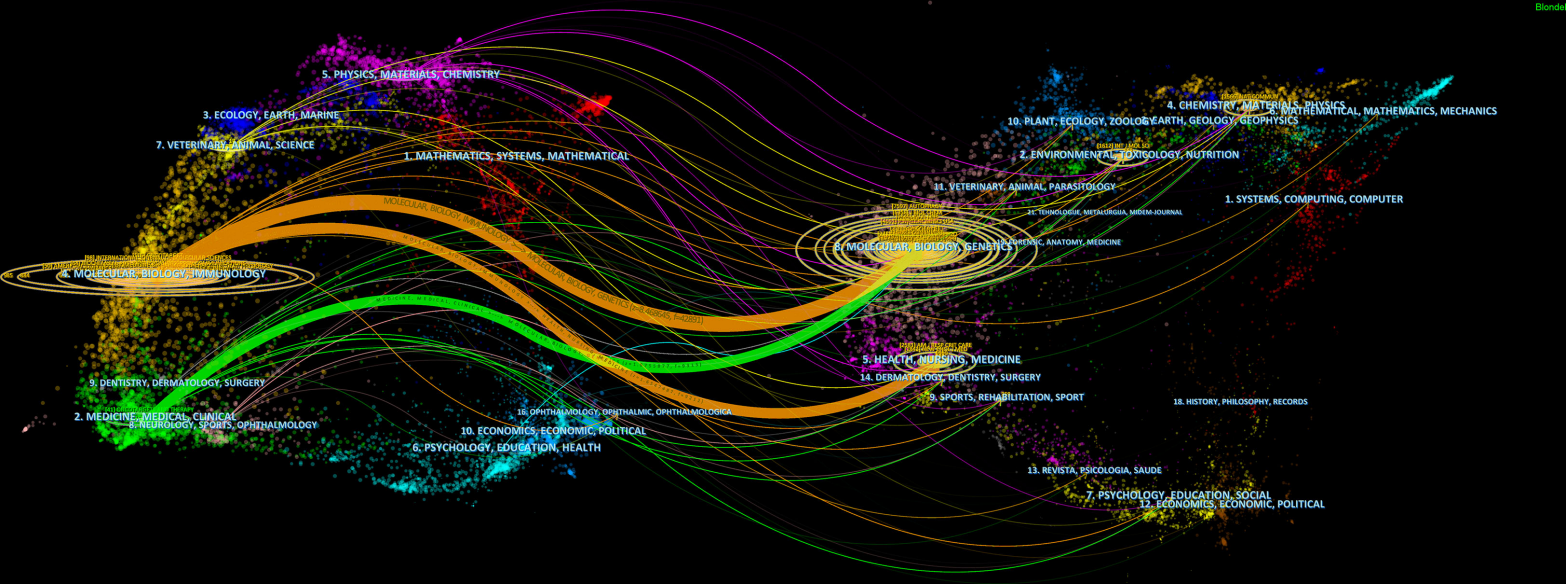
A biplot overlay of journals on autophagy research in lung disease (left and right sides refer to the citing journal areas and the cited journal areas, respectively).

CiteSpace assisted in constructing the subject distribution regarding academic journals by virtue of the biplot overlay function (**Figure 7**). According to the journal biplot overlay, there were mainly two citation paths. Journals publishing articles were basically in medical sciences covering the pharmaceutical, clinical, molecular, biological, and immunological fields, and most cited articles were published in journals in molecular, biological, immunologic, genetic, dermatological, health, nursing, rehabilitation, dental, surgical, sports, neurological, pharmaceutical, and ophthalmological fields.

### Co-cited reference clusters analysis

**Figure 8:**
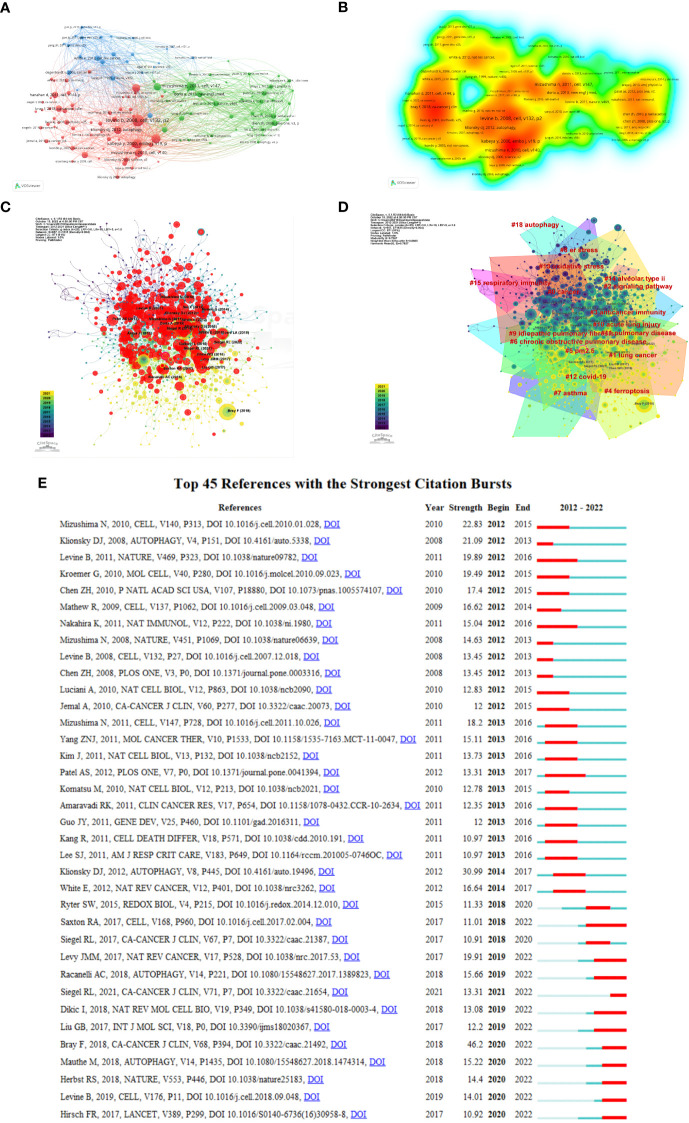
Visualization of co-cited references on autophagy research in lung disease. **(A)** Reference co-citation network. Circles are co-cited literature. **(B)** Co-cited reference density visualization. **(C)** Network visualization diagram of cited references. **(D)** The clustered network map of lung disease autophagy-related co-cited references. **(E)** The 45 references having the highest citation burst.

A co-citation network refers to a reference network simultaneously co-cited by at least one paper. Conceptual clusters were created when a set of manuscripts was cited together repeatedly (27). As shown in **Figures 8A, B**, the most cited article was written by Basant A. Abdulrahman who pointed out that rapamycin, an autophagy stimulator, enhanced the clearance of *C. coronans* by inducing autophagy and significantly reduced the infection of *C. coronans in vitro*.

Rapamycin was found capable of lowering the bacterial burden in the mouse lung *in vitro* and markedly eliminating lung inflammation signs (28). **Figure 8C** demonstrates a network visualization map regarding cited references, with nodes denoting the cited reference, where the connecting lines stand for the co-citation relationship. The different colors varying from purple to yellow mean different years in the range of 2012 to 2021. Node size denotes the number of co-citation number. **Figure 8D** displays the 16 relevant clusters divided by CiteSpace: cancer, lung cancer, signaling pathway, anticancer immunity, ferroptosis, particulate matter (PM2.5), COPD, asthma, ER stress, idiopathic pulmonary, acute lung injury, pulmonary disease, COVID-19, oxidative stress, alveolar type II, respiratory immunity, and autophagy. Citation burst reference refers to the sudden citation increase of specific articles within a certain period, which serves for finding the latest high-profile research topics in relative fields (29). We obtained 674 references with the highest citation burst and selected the top 25 (**Figure 8E**). The blue line denotes the timeline, and the red sections denote the burst interval, respectively, showing the beginning and end of the year, and the burst duration.

### Analysis of keywords

**Figure 9:**
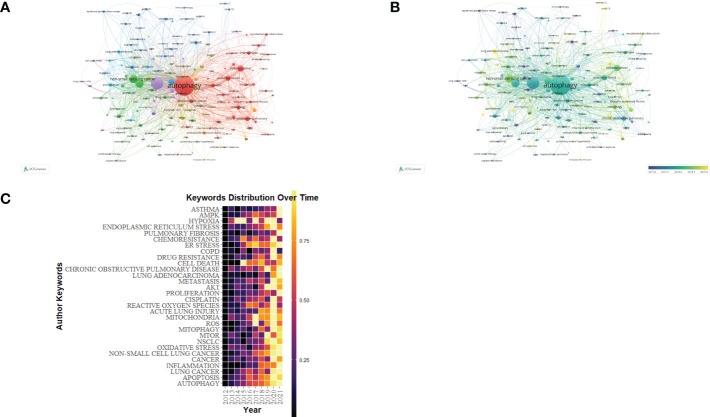
The keyword mapping of autophagy research in lung disease. **(A)** The 155 keywords appearing over 13 times fell into five clusters based on colors: Cluster 1, 2, 3, 4, and 5 are, respectively, red, green, blue, yellow, and purple. The node size denotes the occurrence frequency. **(B)** Keywords visualization based on the APY. Keywords in cluster 4 occurred later than those in cluster 3. **(C)** Yearly occurrences of the top keywords.

Keywords demonstrate a paper’s principal ideas and theme concepts, briefly describing the certain research hot spots. VOSviewer and CiteSpace were used for drawing charts. We identified words that appeared over 13 times in the process of analysis as keywords and finally obtained 155 keywords (**Figure 9A**; **Table 6**). As shown in **Figure 9C**, the closer the keyword was closer to yellow, the more frequently it appeared in the year. According to **Figure 9A**, clusters 1 and 5 were mainly composed of autophagy, apoptosis, and death, which paid attention to relevant mechanisms in the programmed cell death. Cluster 2 stands for “oxidative stress”, “mitochondrial dysfunction”, and “lung disease”. Oxidative stress, the oxidation-antioxidant imbalance in the body, usually causes mitochondrial dysfunction and lung disease. Cluster 3 emphasizes the function of autophagy in lung diseases caused by COVID-19. Clusters 4 and 5 mainly reflect the cellular mechanism and molecular pathway of lung diseases caused by autophagy. In overlay visualization, the color of keywords depends on the average publication year (APY). As shown in **Figure 9B**, the VOSviewer colors all keywords on the basis of APY. The most recent keyword was “sars-cov-2” (cluster 3, APY: 2020.53), followed by “COVID-19” (cluster 3, APY: 2020.45), both closely related to COVID-19. The color division confirms that the most recent keywords in the research field were “pyroptosis“, “immunity”, “COVID-19”, and “lung cancer”, which are proven to be the recent research hot spots.

**Table 6:** Top 20 keywords of publications regarding autophagy research in lung disease based on occurrences.

Rank	Keywords	Occurrences	TLS	Rank	Keywords	Occurrences	TLS
1	Autophagy	1,967	1,734	11	ROS	87	136
2	Apoptosis	757	1,025	12	Mitochondria	85	110
3	Non–small cell lung disease	326	326	13	Reactive Oxygen Species	84	126
4	Lung cancer	309	348	14	Acute lung injury	84	112
5	Inflammation	205	259	15	Cisplatin	75	115
6	Cancer	187	172	16	Proliferation	74	119
7	Oxidative stress	140	203	17	AKT	68	103
8	Chronic obstructive pulmonary disease	117	121	18	Metastasis	67	83
9	MTOR	94	137	19	Macrophages	67	70
10	Mitophagy	89	113	20	Lung adenocarcinoma	64	69

### Analysis of research for autophagy caused by COVID-19

**Figure 10:**
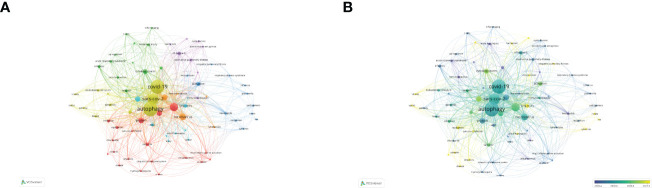
Visualization analysis of autophagy research in lung disease resulted from COVID-19. **(A)** Keyword network mapping. A total of 76 keywords appearing for over two times fell into six clusters based on colors. **(B)** APY-based keyword visualization. Keywords in yellow occurred later than those in blue (color version can be found online).

Following the COVID-19 epidemic outbreak in December 2019, research on lung disease increased markedly in 2020 (**Figure 10A**). Our study screened out and analyzed 76 papers on lung diseases and autophagy related to COVID-19 from the retrieved literature in an attempt to reveal the research focuses and trends in the association between severe lung diseases and autophagy resulting from COVID-19 (**Figure 10B**). The terms inflammation, immunology, cytokine storm, oxidative stress, and macrophages all had the highest frequency as revealed in the keyword visualization analysis, in addition to COVID-19, SARS-COV-2, and lung-related diseases, explaining how autophagy exerted its function in severe lung disease caused by the pathogenesis of COVID-19. In addition, the APY-based keyword color classification by VOSviewer confirmed “inflammation” and “oxidative stress” as the top two recent keywords, meeting previous findings on autophagy in lung diseases. In addition, **Figure 10** shows the involvement of autophagy in the pathogenesis of COVID-19. We also found that the mechanism of anti-inflammatory treatment and immunomodulation therapy for COVID-19–related lung disease was through inhibiting autophagy activation (**Figure 10**).

## Discussion

In this study, we used VOSviewer, CiteSpace, and R to investigate the research dynamics and hot spots of autophagy in lung diseases in the SCI-Expanded. A total of 4,522 publications were obtained from WoSCC. It was found that there was a substantial increase in the number of annual publications and citations during 2012 and 2021, particularly after 2015. China, which published the most papers, has made greater contributions to the study of autophagy in lung diseases. Moreover, nine of the top 10 productive institutions were from China, including China Medical University, Shandong University, and Zhejiang University, indicating that autophagy has drawn increasing attention from Chinese research institutions in the study of lung diseases recently. However, the H-index and G-index of the US were relatively high compared with that of China. The US ranked first among all countries in both H-index and G-index, demonstrating that the quality of documents from the US is high. Despite China’s rapid development and dominant position in the field, it is necessary to improve the collaboration and academic influence in regions. The international distribution of current research in this field is imbalanced.

Choi Augustine M. K. is the most published and cited author in this field. He has long studied the relationship between acute and chronic lung diseases and molecular, cellular, and genetic triggers. He proposed that lung disease may be associated with a pathological accumulation of mutated α1-antitrypsin and that autophagy may be a clearance mechanism for this disease (3). A proper method for monitoring autophagy has been established by Klionsky et al., who suggested that autophagy defects could be induced in favor of lung tumor formation by modulating the tumor microenvironment (28). Noboru Mizushima is also a very influential writer whose works are widely cited. In 1998, Noboru Mizushima reported the homologous gene of Atg12 in human and clarified autophagy (cell self-digestion) as a cellular pathway capable of assisting in degrading proteins and organelles, which is deeply related to human diseases and physiology (30). Yoshinori OhSumi, winner of the 2016 Nobel Prize in Physiology or Medicine, made excellent achievements regarding the function had by autophagy (31). The results of his work have already assisted us in better understanding the function and the effect of autophagy on disease and health (32). The above authors are well respected as they more comprehensively elucidate the mechanism regarding autophagy and contribute to the prevention of lung diseases.

Usually, there is a close relation between the impact of a journal and the impact of its articles (33). Amid the top 10 journals that had the most published papers, AUTOPHAGY exhibited the highest IF (16.016), which was first published in 2005 and is now a leading journal in the field. ONCOTARGET was the most prolific journal, with an IF of 4.147. The journal is sponsored by the US and focuses on articles related to cancer, tumors, autophagy, and cellular immunity. In addition, most active journals are in the professional category. Several multidisciplinary journals, such as CELL DEATH DIS and BIOMED PHARMACOTHER, have also published high-quality studies investigating autophagy in lung disease, which is an emerging and developing research topic and involves molecular biology, cell biology, biochemistry, and medicine. Journals of various disciplines have published advances in such research topic, demonstrating the wide attention paid to lung disease pathogenesis and treatment. In addition, multidisciplinary journals have a wider readership (34), more benefiting interdisciplinary cooperation.

As the research continuously progresses, researchers have revealed the signal pathway that affects different mechanisms. On the basis of keyword mapping, oxidative stress critically impacts the pathogenesis of autophagy, regulating the progression of related lung diseases. The activation of autophagy in response to oxidative stress is mainly for avoiding cell apoptosis (35, 36), In contrast, autophagy inhibition increase oxidative stress damage and even cell death. When ROS is elevated, the HIF-1α transcription factor, p53, FOXO3, and Nrf2 will be activated, which then triggers BNIP3 and NIX, TIGAR, LC3, and p62 (37) to transcribe. The oxidized ATG4 can facilitate LC3 lipidation to initiate autophagy and autophagosome formation. According to a recent report, angiotensin II (Ang II) vasoconstriction can induce autophagy, revealing the ability of Ang II to form autophagic vesicles that contain LC3 and to elevate the ATG12–ATG5, ATG7, and ATG4, thereby simulating the elongation of phagophores (38). As for human glomerular mesangial cells, oxidative stress triggered by Ang II can use mTORC1 signaling for enhancing autophagy, leading to early-stage senescence (39). ROS was found capable of regulating autophagy through pathways dependent of mTOR in the cytoplasm (40, 41). ROS inhibits the PI3K-Akt-mTOR pathway or activates AMPK for suppressing the mTOR signaling pathway, thereby resulting in autophagy activation (42). The increase in ROS oxidizing phosphatase and tensin homolog helps inhibit Akt-mTOR activity (43). Similarly, excess H_2_O_2_ is capable of triggering autophagy dependent of AMPK, coupled with weakened mTORC1 activity (44). Thioredoxin-interacting protein (TXNIP) takes charge of regulating the autophagy of rats with diabetic nephropathy *via* the mTOR signaling pathway (45). In lung diseases, hypoxia caused by the weakened lung functions primarily stimulates the autophagy induction (45). As for the primary human lung vascular endothelial and smooth muscle-cells, hypoxia could induce BECN1 expression, activate LC3, promote autophagosome formation, and stimulate the autophagic flux, thereby protecting mice from pulmonary vascular disease (46). Therefore, this provides a clear path for us to further explore the autophagy-lung disease relationship.

When we talk about the impact of autophagy on various lung diseases, COVID-19 is an unavoidable topic associated with autophagy-mediated lung disease (**Figure 9A**). The SARS-CoV-2 virus causing COVID-19 exhibits a strong infectivity and can trigger cytokine-storm, inducing acute respiratory distress syndrome (ARDS)–like lung injury that can develop into pneumonia and severe lung damage, causing high risk of mortality, especially in susceptible populations (47–49). According to recent studies, SARS-CoV-2 is capable of restricting autophagy, which, as anticipated, is an underlying mechanism for serious COVID-19 lung disease as it impairs the viral clearance ability and causes immune dysfunction (50, 51). Recently, researchers have revealed the stronger affinity exhibited by NSP6 protein of SARS-CoV-2 in binding with the ER (52). Because of the genetic alternation, virus is capable of restricting autophagy through damaged autophagosome processing, where viral particles can be less degraded by lysosome (52). In addition, relying on PLP2 overexpression in SARS-CoV and MERS-CoV cell lines, the virus is capable of preventing autophagolysosomal formation and suppressing autophagy flux, which may serve for inhibiting autophagy in SARS-CoV-2 (53). These studies reveal the potential ways for SARS-CoV-2 to inhibit autophagy infection or avoid the pathogenic clearance of host cells, meanwhile restricting the sufficient immune response, which is similar to other types of coronaviruses, and need to be further investigated. Eliminating viruses by autophagy (also called virophagy) can well serve for various viral infections (54, 55). Despite the various ways through viruses get access to cells, autophagy augmentation can promote virus clearance, thus reducing the viral load from a strategic perspective (56, 57). To prove this from a conceptual perspective, some recent studies have investigated how the three different drugs (spermidine, MK02206, and niclosamide) induced by autophagy restrict the propagation of SARS-CoV-2 (58). Autophagy induction together with relevant overall immunity upregulation assists in combating the exacerbations, which may prove to be a strategy to enhance immunity and prevent COVID-19 infection (59). Autophagy induction can enhance immunity, restrict viral load, support SARS-CoV-2 clearance, and simultaneously assist in treating COVID-19 infection and preventing negative results, although further investigation is required to confirm these effects.

The pathogenesis regarding severe COVID-19 mediated by SARS-CoV-2 is pointed out to be associated with the activation of many pro-inflammatory cytokines that constitute the cytokine storm, resulting in a hyper-inflammatory state. In addition to destroying the lung and COVID-19–related ARDS, such inflammatory response is also capable of damaging the cardiovascular, nervous, and gastrointestinal systems and bringing into direct and long-term outcomes (60). To support these findings, studies have revealed how autophagy affects the inflammatory response in organ systems including the lung (61, 62). Accordingly, autophagy induction is capable of attenuating lung inflammation in the event of being exposed to pathogens. Hence, except for infection, using autophagy induction for restricting the inflammatory response has huge potentials for treating COVID-19 and decreasing relevant morbidity and mortality.

In this study, we employed the bibliometric approach for visualizing the research on autophagy-mediated lung diseases, thereby gaining a better understanding about the hot spots and trends in this field. Nevertheless, it suffers many limitations (1). The study only included English articles and reviews from the SCI-Expanded. (2) Currently, the limitation of software-based scientific measurement added the difficulty in combining at least two databases for the analysis. Hence, the study only adopted the WoSCC database for the screening and might have missed the other related studies in the literature. Therefore, more databases should be employed for a more rigorous analysis in the future. (3) Open-source journals may have an impact on both citation volume and publication volume. (4) The combination of CiteSpace and VOSviewer for literature analysis may miss information during analysis. Specifically, VOSviewer keyword analysis clustering might not have been able to extract key information, because color was only used to divide the range.

## Conclusions

According to bibliometric analysis, the research on autophagy in the field of lung diseases has broad prospects and rapid development. We identified the major contributors, regions, and publications in the research area. China published the largest NP and the US made more huge breakthroughs. The latest studies have been published and new progression regarding the field has been made on AUTOPHAGY and ONCOTARGET. Recently, oxidative stress has been revealed to have significant impact on the pathogenesis of autophagy-mediated lung diseases. In addition, the mechanism of autophagy induction may prove crucial as a treatment strategy for COVID-19 over classical antivirals and provide a tool to end the pandemic. Attenuating the progression of COVID-19–associated lung diseases by inhibiting autophagy may become a new research hotspot in this field.

## Data availability statement

The original contributions presented in the study are included in the article/supplementary material. Further inquiries can be directed to the corresponding authors.

## Author contributions

FL, YC and WM did the bibliometrics analysis and wrote the manuscript. HZ, ZX, SH, XS, ML, JW and WZ participated in the experimental design and manuscript writing. SW and XL designed this study and organized the manuscript writing. All authors contributed to the article and approved the submitted version.
